# The new era of add-on asthma treatments: where do we stand?

**DOI:** 10.1186/s13223-022-00676-0

**Published:** 2022-05-21

**Authors:** William J. Calhoun, Geoffrey L. Chupp

**Affiliations:** 1 Divisions of Pulmonary, Critical Care, and Sleep Medicine, and Allergy/Immunology; and Institute for Translational Sciences, University of Texas Medical Branch, 4.116 John Sealy Annex, 301 University Blvd, Galveston, TX 77555-0568 USA; 2 Division of Pulmonary, Critical Care, and Sleep Medicine, Yale Center for Asthma and Airway Disease, Yale University School of Medicine, New Haven, CT USA

**Keywords:** Add-on, Add-on therapy, Biological therapy, Severe asthma, Tiotropium bromide

## Abstract

Globally, a small proportion (5–12%) of asthma patients are estimated to have severe disease. However, severe asthma accounts for disproportionately high healthcare resource utilization. The Global Initiative for Asthma (GINA) management committee recommends treating patients with asthma with inhaled corticosteroids plus long-acting β_2_-agonists and, when needed, adding a long-acting muscarinic receptor antagonist or biologic agent. Five biologics, targeting different effectors in the type 2 inflammatory pathway, are approved for asthma treatment. However, biologics have not been compared against each other or add-on inhaled therapies in head-to-head clinical trials. As a result, their positioning versus that of current and anticipated small-molecule strategies is largely unknown. Furthermore, with the emergence of biomarkers for predicting response to biologics, a more personalized treatment approach—currently lacking with inhaled therapies—may be possible. To gain perspective, we reviewed recent advances in asthma pathophysiology, phenotypes, and biomarkers; the place of biologics in the management and personalized treatment of severe asthma; and the future of biologics and small-molecule drugs. We propose an algorithm for the stepwise treatment of severe asthma based on recommendations in the GINA strategy document that accounts for the broad range of phenotypes targeted by inhaled therapies and the specificity of biologics. In the future, both biologics and small molecules will continue to play key roles in the individualized treatment of severe asthma. However, as targeted therapies, their application will continue to be focused on patients with certain phenotypes who meet the specific criteria for use as identified in pivotal clinical trials.

**Supplementary Information:**

The online version contains supplementary material available at 10.1186/s13223-022-00676-0.

## Background

**Fig. 1:**
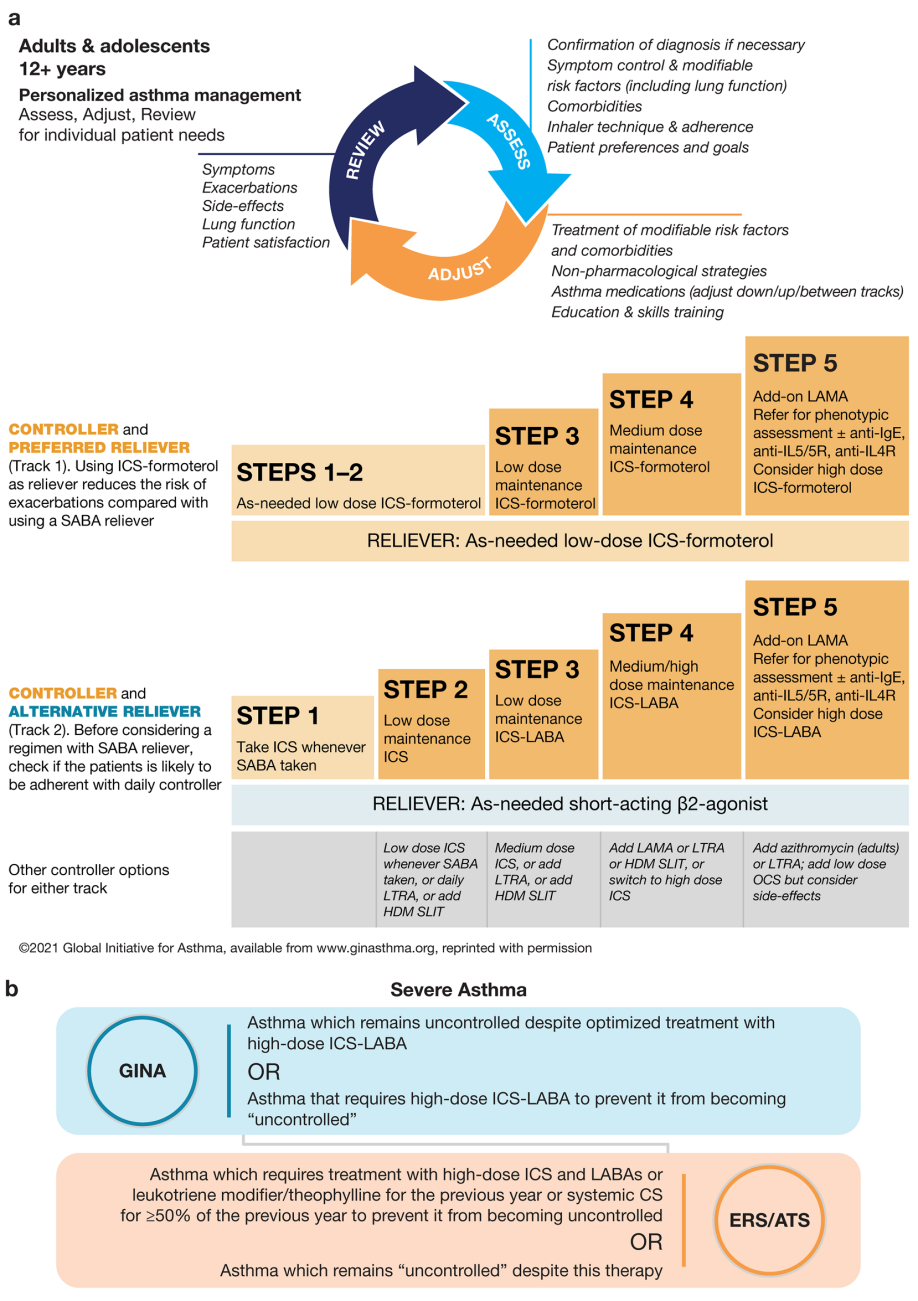
**a** GINA recommended stepwise asthma treatment. **b** Severe asthma definition [1, 2]. *ATS* American Thoracic Society, *BDP* budesonide propionate, *CS* corticosteroids, *ERS* European Respiratory Society, *FEV*_*1*_ forced expiratory volume in 1 s, *GINA* Global Initiative for Asthma, *HDM* house dust mite, *ICS* inhaled corticosteroids, *IgE* immunoglobulin E, *IL* interleukin, *LABA* long-acting β_2_-agonist, *LAMA* long-acting muscarinic antagonist; *LTRA* leukotriene receptor antagonist, *OCS* oral corticosteroids; *SABA* short-acting β_2_-agonist; *SLIT* sublingual immunotherapy

Severe asthma is defined by the ERS/ATS guidelines [1] as asthma which requires treatment with high-dose inhaled corticosteroids (ICS) and long-acting β_2_-agonists (LABAs) or leukotriene modifier/theophylline for the previous year or systemic corticosteroids for ≥ 50% of the previous year to prevent it from becoming uncontrolled or which remains uncontrolled despite this therapy. The Global Initiative for Asthma (GINA) [2] defines severe asthma as asthma which remains uncontrolled despite optimized treatment with high-dose ICS-LABA, or that requires high-dose ICS-LABA to prevent it from becoming uncontrolled (Fig. 1). “Uncontrolled” asthma is characterized by the presence of poor symptom control, frequent severe exacerbations, serious exacerbations, or airflow limitation [1].

Of the global asthma population, approximately 5% to 12% [3–5] and < 4% [3, 6] are estimated to have severe asthma and severe, uncontrolled asthma, respectively; however, the true prevalence of severe uncontrolled asthma may be substantially greater as there is no consistent definition across studies. Although the proportion of patients with severe asthma may seem low, millions of individuals are afflicted, so the impact on society, associated personal and socioeconomic burdens are high [6, 7]. Persistent symptoms, frequent exacerbations, impaired quality of life (QoL), and eventual loss of lung function are common challenges faced by patients with severe asthma [7]. Furthermore, results of an observational study of patients with persistent asthma in a real-world, US managed-care setting led investigators to conclude that patients with severe asthma require more intensive therapy, greater attention to adherence and comorbidities, and more specialist care than patients with non-severe asthma [6].

## Pathophysiology and inflammatory pathways in asthma

**Fig. 2:**
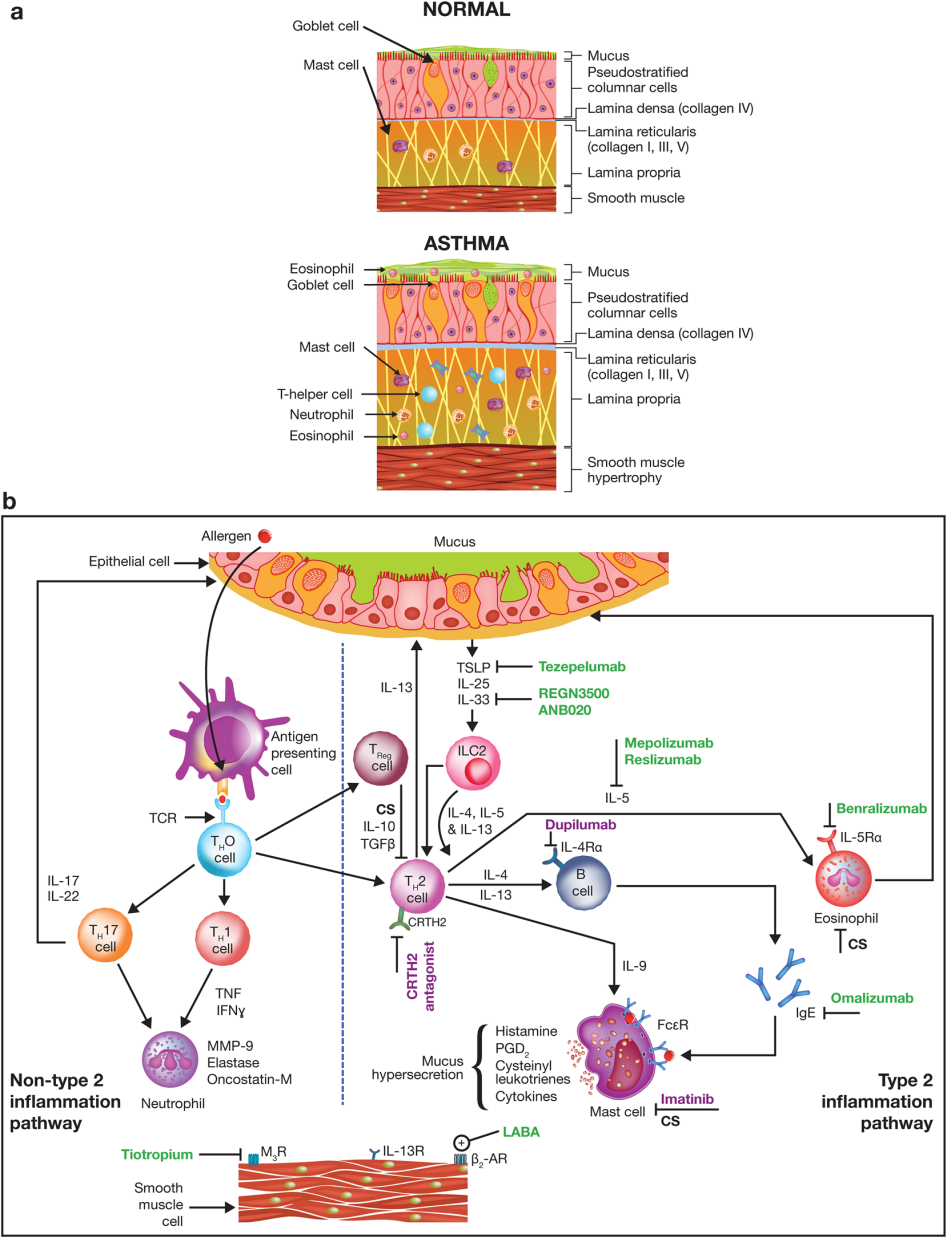
**a** Cross-section and histology of airways in a normal person and a patient with asthma. **b** Pathways of inflammatory responses, biomarkers, and mechanism of action of various therapeutic agents. *β*_*2*_*-AR* β_2_-adrenergic receptor, *CS* corticosteroids, *CRTH2* chemoattractant receptor-homologous molecule expressed on Th2 cells, *FcεR* Fcε receptor, *IFN* interferon, *Ig* immunoglobulin, *IL* interleukin, *ILC2* innate lymphoid cell type 2, *LABA* long-acting β_2_-agonist, *MMP-9* matrix metallopeptidase 9, *M*_*3*_*R* muscarinic receptor type 3, *PGD*_*2*_ prostaglandin D_2_, *TCR* T-cell receptor, *TGF* tumor growth factor, *TNF* tumor necrosis factor, *Th* T helper, *TSLP* thymic stromal lymphopoietin

Asthma is characterized by variable obstruction, hyperresponsiveness, and inflammation of the airways. Airway obstruction results from contraction of the airway smooth muscles, mucosal inflammation, and airway remodeling involving structural changes, such as collagen deposition, airway smooth muscle hyperplasia and hypertrophy, and excess mucus production (Fig. 2). Severe asthma pathophysiology involves a greater degree of incompletely reversible airflow limitation, more severe airway remodeling, marked thickening of the airway wall, and excessive airway narrowing upon stimulation of airway smooth muscle contraction [8, 9].

Inflammation in asthma is heterogeneous in nature. Type 2 (T2) inflammation is characterized by the release of interleukin (IL)-4, IL-5, and IL-13 [8]. IL‐4 induces B-cell class switching to produce immunoglobulin E (IgE), which binds to high-affinity surface receptors on mast cells; degranulation occurs following IgE crosslinking with antigens. IL‐13 induces mucus hypersecretion and regulates epithelial cell function. IL-5 is associated with epithelial changes, leading to increased eotaxin expression; IL-5 promotes the proliferation, maturation, and survival of eosinophils, and is associated with airway eosinophilia [8, 10].

A T2 inflammatory response is usually initiated by type 2 cytokines, released by a subpopulation of CD4+ T cells (T-helper type 2 [Th2] cells), or innate lymphoid cells type 2 (ILC2s), which are also a major source of type 2 cytokines [11, 12]. ILC2s are activated by alarmins or epithelial cell-derived cytokines (thymic stromal lymphopoietin [TSLP], IL-33, and IL-25), which are released upon exposure to infectious agents and allergic stimuli [11]. Alarmins drive tissue inflammation by promoting type 2 cytokine release from ILC2s and development of Th2 cells. Thus, alarmins can activate both innate (ILC2s) and adaptive (Th2 cells) immune cells and are thought to be a key link between innate and adaptive immune responses in T2 inflammation [13].

The prostaglandin D_2_ (PGD_2_) pathway is also involved in T2 inflammation [14]. Mast cell activation and mast cell-derived PGD_2_ is increased in severe asthma, which activates mast cells and ILC2s [8].

Non-T2 (or type 1) inflammation is associated with non-T2 inflammatory pathways. Neutrophilic inflammation, as demonstrated by neutrophils in induced sputum, may be seen in a subset of patients with non-T2 asthma and is likely driven by CD4+ T cells—T-helper type 1 and type 17 cells [15]. Paucigranulocytic inflammation, characterized by the absence of increased eosinophils and neutrophils in induced sputum, is the cause of airflow limitation in the absence of cellular inflammation in the pathways and may be associated with “stable” asthma [16]. Paucigranulocytic asthma is often associated with controlled as opposed to stable asthma but there may be a small subset of patients with a phenotype whereby paucigranulocytic asthma is related to structural changes due to hyperresponsiveness [17]. Based on studies in animal models, airway obstruction in paucigranulocytic asthma has been linked to structural changes in airway smooth muscle via upregulation of asthma-specific genes [16].

Accumulating evidence, including increased neutrophilic inflammation in severe asthma [18] and increased neutrophil counts during asthma exacerbations [19], suggests that neutrophils may be associated with some types of severe asthma. This is supported by studies that showed neutrophil-generated matrix metalloprotease-9 and elastase promote airway remodeling, and the neutrophil-released cytokine, oncostatin-M, affects the epithelial barrier function in patients with severe asthma [15].

Airway hyperresponsiveness, a consistent feature of asthma, involves an exaggerated or excessive bronchoconstrictor response to stimuli. Although the mechanisms leading to airway hyperresponsiveness are poorly understood, increased activity of parasympathetic cholinergic pathways, in part, may contribute to airway hyperresponsiveness, independent of the underlying inflammation [20, 21]. Thus, anticholinergic therapies that target muscarinic receptors are effective. Sympathetic control in the airways is mediated via β_2_-adrenoreceptors expressed on airway smooth muscle cells and is the basis of the efficacy of short-acting β_2_-agonists and LABAs.

An additional video file illustrates the pathophysiology of asthma (see Additional file 1: Video S1).

## Asthma phenotypes

Most early attempts to define asthma phenotypes were one dimensional and restricted to age of onset and atopy. A multidimensional approach has since been used to identify subgroups of patients with consistent patterns of clinical disease [22–25]. In a population of asthma patients receiving specialty care, four phenotypic clusters were identified: early-onset asthma, obesity-related asthma, symptom-predominant asthma with minimal eosinophilia, and the predominant eosinophilic late-onset asthma with few symptoms [23]. Because of the discordance between underlying inflammation and symptom presentation, the authors posited a role for examining underlying eosinophilic airway inflammation when deciding treatment versus the traditional approach of symptom-led ICS titration [23].

Multiple asthma phenotypes representing the spectrum of asthma severity (early-onset allergic asthma, late-onset severe asthma, and severe asthma with chronic obstructive pulmonary disease characteristics) were identified in a cohort of patients in the Severe Asthma Research Program (SARP) [22]. Among the patients who underwent sputum induction, four sputum inflammatory cellular patterns were identified based on median percentages of eosinophils (2%) and neutrophils (40%) [26]. Patients in clusters A and B (259/423 [61%]) had mild-to-moderate, early-onset asthma and either an eosinophil-predominant (≥ 2% eosinophils) or paucigranulocytic (< 2% eosinophils and < 40% neutrophils) sputum inflammatory cellular pattern. In contrast, most patients (83%) in clusters C and D (164/423 [39%]) with moderate-to-severe asthma had sputum neutrophilia, alone or with concurrent sputum eosinophilia. These results highlight the marked differences in inflammatory cell involvement across different clinical asthma phenotypes indicating that clinical heterogeneity is driven by pathobiological heterogeneity.

In a different cohort of patients from the Unbiased BIOmarkers in PREDiction of Respiratory Disease Outcomes (UBIOPRED) Consortium [27], four reproducible and stable clusters were identified: well-controlled, moderate-to-severe asthma; late-onset severe asthma in smokers and ex-smokers with chronic airflow obstruction; asthma in nonsmokers with chronic airflow obstruction; and obesity-related, uncontrolled, severe asthma in patients with increased exacerbations, but normal lung function. Although sputum neutrophil counts were similar across the clusters, sputum eosinophil counts were higher in the severe asthma clusters than in the moderate-to-severe asthma cluster.

Collectively, these studies suggest that asthma can be grouped into clinical phenotypes that are driven by several different endotypes [24]. The main inflammatory phenotypes are Th2 high (eosinophilic subtype) and Th2 low (noneosinophilic/paucigranulocytic and neutrophilic subtypes). Transcriptomic signatures may help better characterize these subtypes. Noninvasive analysis of sputum gene expression enabled identification of transcriptomic endotypes of asthma clusters that correlate with distinct clinical phenotypes of severe asthma [28]. Elucidating the underlying asthma endotypes and associated phenotypes may lead to improved precision-based medicine. Identification of patients within each phenotype using novel specific biomarkers and clinical symptoms may enable clinicians to use the right medication for the right patient.

## Overview of current treatment recommendations

Established treatment options for severe asthma include small molecules such as ICSs, LABAs, the long-acting muscarinic antagonist (LAMA) tiotropium, and leukotriene receptor antagonists (LTRAs). Global guidance is provided by the GINA committee [2] and US-based guidance by the National Heart, Lung, and Blood Institute (NHLBI) [9, 29]. Per the stepwise approach outlined in the GINA strategy document [2], medium-dose maintenance ICS plus formoterol is recommended as the preferred treatment at GINA Step 4 for moderate-to-severe asthma (Fig. 1). Although LABA-based asthma medications had a black-box warning for asthma-related death for over a decade, the US Food and Drug Administration (FDA) removed the black-box warning from ICS + LABA drug labels in 2017 based on findings from four clinical trials involving 41,297 patients [30]; drug labels for single-ingredient LABA medications retain the warning. Tiotropium, the only LAMA approved for use in patients (aged ≥ 6 years) with asthma [31], may be considered as add-on therapy for children, adolescents, or adults with a history of exacerbations at GINA Step 4. Considering high-dose ICS plus formoterol, with add-on LAMA or biologics, are the preferred treatments at GINA Step 5 [2]. The NHLBI recommends daily ICS + LAMA, as early as Steps 3 and 4, as an alternative therapy, and as a preferred therapy at Step 5 for the management of persistent asthma in patients aged > 12 years [29].

However, before stepping up treatment, the presence of severe asthma—versus uncontrolled asthma caused by incorrect inhaler technique and/or poor adherence, or confounding factors and comorbidities—needs to be confirmed [1, 2]. GINA has provided general recommendations for managing severe asthma, all accompanied by caveats and limitations [32]; strategies include: optimizing the ICS + LABA dose in appropriate patients; low-dose maintenance oral corticosteroids (OCS), with strategies to minimize associated long-term side-effects; add-on treatment with tiotropium or macrolide; phenotype-guided (e.g., severe allergic, aspirin exacerbated) add-on treatments; and nonpharmacological approaches, such as bronchial thermoplasty.

Besides patient characteristics and phenotypes, patient’s preferences and practical issues (inhaler technique, adherence, and cost to patient) must also be considered while making treatment decisions.

## Add-on asthma treatments

### Use of LAMAs in asthma

Tiotropium reduces airflow obstruction by antagonizing muscarinic type-3 receptors on airway smooth muscles and submucosal glands, leading to bronchodilation and decreased mucus secretion (Fig. 2; see Additional file 1: Video S1) [33, 34]. Tiotropium, when added to background treatment of at least an ICS or ICS + LABA, significantly improved lung function and reduced asthma exacerbation rates (Table 1) in adult patients with moderate-to-severe asthma [35–38]. In a real-world cohort of patients with asthma initiated on ICS + LABA, compared with increasing the ICS plus LABA dose, add-on tiotropium significantly decreased all-cause and asthma-related emergency department visits and hospitalizations, the risk and rate of exacerbations, and the number of short-acting β2-agonist refills [39]. Evidence from other real-world studies supports data from randomized clinical trials and showed that add-on tiotropium resulted in improved clinical outcomes as well as reduced emergency department visits and hospitalizations in severe asthma patients [40, 41]. Of note, in a systematic review and meta-analysis of 15 randomized clinical trials (7122 patients), add-on tiotropium and ICS were associated with a significantly lower risk (risk ratio: 0.67 [95% CI 0.48, 0.92]) of asthma exacerbations than with placebo and ICS [42]; moreover, the treatment benefits from a LAMA were comparable to those from a LABA. Overall, these data suggest that treatment with ICS + LAMA can help reduce the risk of future exacerbations in patients inadequately controlled on ICS alone [43].

**Table 1:** Approved add-on asthma treatments

Category	Drug, age	Approved dosing	Efficacy	Safety	Specificity
Anti-muscarinic	Tiotropium Respimat®, ≥ 6 years [31, 35–38]	2.5 µg (2 × 1.25 µg/puff) once daily (US FDA-approved dose) or 5 µg (2 × 2.5 µg/puff) once daily	• Improved peak FEV_1(0–3 h)_ and trough FEV_1_ • Increased time to first severe exacerbation	Common AEs: nasopharyngitis, headache, bronchitis, and upper respiratory tract infection	Useful across all phenotypes of GINA Step 4/5 asthma and severe, uncontrolled asthma
Anti-IgE	Omalizumab, ≥ 6 years [50, 59–63]	75–375 mg SC Q2W or Q4W; (varies by serum total IgE level and weight)	• Reduced exacerbation rate, emergency visits, and rescue medication use • Improved FEV_1_, ACT, AQLQ, and IGETE scores	Common AEs: asthma, upper or lower respiratory tract infection, nasopharyngitis, sinusitis, bronchitis, and headache	Indicated in patients with positive skin test or in vitro reactivity to a perennial aeroallergen and serum total IgE levels: 30–700 IU/mL Useful in patients with Th2-high phenotype
Anti-IL-5	Mepolizumab, ≥ 6 years [48, 70–72]	100 mg SC Q4W	• Reduced asthma exacerbation risk and blood eosinophil counts • Improved FEV_1_ and SGRQ and ACQ-5 scores	Common AEs: headache and nasopharyngitis	Useful in patients with baseline blood eosinophil counts ≥ 150 cells/µL
	Reslizumab, ≥ 18 years [47, 73–76]	3 mg/kg Q4W IV infusion over 20–50 min	• Improved FEV_1_, FVC, and FEF_25–75%_, ACQ, and AQLQ scores • Reduced frequency of asthma exacerbations and rescue medication use	Common AEs: worsening of asthma, headache, nasopharyngitis, upper respiratory tract infection, sinusitis, influenza, and headache	Useful in patients with baseline blood eosinophil counts ≥ 400 cells/µL
	Benralizumab, ≥ 12 years [49, 80, 81]	30 mg Q4W SC for the first three doses, followed by Q8W thereafter	• Reduced annual asthma exacerbation rate, blood eosinophil counts, ACQ-6 scores, and corticosteroid dose • Improved prebronchodilator FEV_1_	Common AEs: worsening asthma, nasopharyngitis, and upper respiratory tract infection	Useful in patients with baseline blood eosinophil ≥ 300 cells/µL
Anti-IL-4Rα	Dupilumab, ≥ 12 years [51, 82]	Initial dose (600 or 400 mg), followed by 300 or 200 mg given every other week	• Reduced annual severe asthma exacerbations rate and oral glucocorticosteroid use • Increased FEV_1_	Transient eosinophilia observed	Useful in patients with moderate-to-severe asthma with baseline blood eosinophils ≥ 300 cells/µL or with oral corticosteroid-dependent asthma
Anti-TSLP	Tezepelumab, ≥ 12 years [53]	210 mg SC Q4W	• Reduced annual asthma exacerbations • Reduced exacerbations, which required emergency room visits and/or hospitalization • Improved FEV_1_	Common AEs: pharyngitis, arthralgia, and back pain	Useful in patients with severe asthma irrespective of their phenotype (e.g., eosinophilic or allergic) or biomarker limitation

*ACT* Asthma Control Test, *ACQ* Asthma Control Questionnaire, *AE* adverse event, *AQLQ* Asthma Quality of Life Questionnaire, *FDA* Food and Drug Administration, *FEF*_*25–75%*_ forced expiratory flow between 25 and 75% of FVC, *FEV*_*1*_ forced expiratory volume in 1 s, *FEV*_*1(0–3 h)*_ forced expiratory volume within 3 h after dosing, *FVC* forced vital capacity, *IGETE* Investigator’s Global Evaluation of Treatment Effectiveness, *IgE* immunoglobulin E, *IL* interleukin, *IV* intravenous, *SC* subcutaneous, *Q2W* once every 2 weeks, *Q4W* once every 4 weeks, *Q8W* once every 8 weeks, *SGRQ* St. George’s Respiratory Questionnaire, *Th* T helper, *TSLP* thymic stromal lymphopoietin, *US* United States

Improvements in lung function, risk of exacerbations, and symptom control with tiotropium versus placebo were independent of patients’ underlying eosinophil counts or IgE levels [44, 45]. Moreover, add-on tiotropium was considered cost-effective compared with standard of care or an anti-IgE biologic (omalizumab) at a willingness-to-pay threshold of $50,000/quality-adjusted life year in a recent US-based, cost-effectiveness study [46]. Based on the evidence, NHLBI guidelines [29], and GINA recommendations [2], tiotropium, the only LAMA approved for the treatment of asthma, should be added to ICS or ICS + LABA before considering a biologic as an add-on therapy.

### Add-on biologics

FDA-approved biologics include omalizumab (anti-IgE), mepolizumab (anti-IL-5), reslizumab (anti-IL-5), benralizumab (anti-IL-5 receptor), dupilumab (anti-IL-4/IL-13 receptor), and tezepelumab (Table 1; see Additional file 1: Video S1) [47–53]. Tezepelumab, which targets TSLP, was recently approved by the FDA for the add-on maintenance treatment of adult and pediatric patients aged ≥ 12 years with severe asthma [53]. Tezepelumab was evaluated in phase 3 trials, such as the NAVIGATOR trial [54], which showed tezepelumab reduced exacerbations in patients with both high and low baseline blood eosinophil count, and improved asthma control, lung function, and health-related QoL in patients with severe, uncontrolled asthma. Among patients treated with medium to high doses of ICS + LABA, tezepelumab reduced rates of clinically significant asthma exacerbations compared with placebo, independent of baseline blood eosinophil counts [55].

Head-to-head clinical trials between biologics are lacking and indirect comparison of biologics has provided conflicting results, presumably because patient populations are not comparable across studies with respect to demographics and clinical characteristics, such as age, lung function, and eosinophil counts. In a network meta-analysis, benralizumab, dupilumab, mepolizumab, and reslizumab were associated with improvements in lung function (forced expiratory volume in 1 s [FEV_1_]), asthma control, and asthma-related QoL to varying degrees, although only reslizumab and dupilumab were found to significantly reduce asthma exacerbation rates [56]. In a global meta-analysis of randomized control trials of mepolizumab, reslizumab, and benralizumab, no clear superiority was observed [57]. However, in an indirect treatment comparison of these three biologics, where patients were stratified by baseline blood eosinophil count, mepolizumab was associated with significantly greater improvements in exacerbations and asthma control [58].

### Biologics targeting IgE

Omalizumab, a monoclonal antibody that binds with IgE, is indicated for moderate-to-severe persistent asthma in patients aged ≥ 6 years with a positive skin test or in vitro reactivity to a relevant perennial aeroallergen and symptoms that are inadequately controlled with ICSs. Omalizumab treatment of patients with poorly controlled severe asthma and total serum IgE levels of 30 to 700 IU/mL improved asthma control, reduced exacerbations, and reduced or did not increase use of other medications (Table 1) [59–63]. Although omalizumab is indicated for use within this IgE-level range, there is some anecdotal evidence [64, 65] indicating that it can be successfully used outside of this range. Strong evidence exists for the use of omalizumab in patients with Th2-high asthma phenotype. In post hoc analyses of pooled data from phase 3 trials, omalizumab reduced exacerbations in patients with high blood eosinophil counts (≥ 260 or ≥ 300 cells/μL) [66, 67]. Furthermore, the difference in exacerbation frequency between omalizumab and placebo groups was greater in patients with moderate-to-severe persistent asthma with high blood eosinophil counts and high fractional exhaled nitric oxide (FeNO) concentrations than their low subgroup counterparts in the EXTRA study [66]. However, real-life data from the STELLAIR study suggest similar omalizumab effectiveness in patients with blood eosinophil counts of ≥ 300 cells/µL and < 300 cells/µL [68].

### Biologics targeting IL-5 or IL-5 receptor

Mepolizumab and reslizumab target IL-5, which promotes the recruitment of eosinophils from the bone marrow and their subsequent proliferation [69]. Mepolizumab and reslizumab are indicated as add-on maintenance treatments for severe eosinophilic asthma in patients aged ≥ 6 years and adults aged ≥ 18 years, respectively (Table 1) [47, 48, 70–76]. Unlike mepolizumab, which is administered subcutaneously, reslizumab is only approved for intravenous administration [47]. The primary endpoints were not reached in phase 3 trials of subcutaneously administered reslizumab [77]. Both drugs are efficacious in eosinophilic asthma; however, the blood eosinophil count thresholds used in their respective pivotal trials were significantly different. In phase 2/3 mepolizumab trials, the thresholds used were blood eosinophil count of ≥ 150 cells/μL at screening or ≥ 300 cells/μL in the year prior to enrollment; ≥ 400 cells/μL was used in reslizumab trials [72–76, 78]. Of note, reslizumab has a black-box warning on anaphylaxis, which was reported in 0.3% enrolled in placebo-controlled clinical trials [47].

Benralizumab targets the IL-5 receptor α-subunit, thereby preventing binding of IL-5 to its receptor, depleting eosinophils and basophils [79]. It also enhances antibody-dependent, cell-mediated cytotoxicity as a consequence of its afucoyslation [49]. Benralizumab is indicated for the add-on maintenance treatment of patients with severe asthma aged ≥ 12 years, and with an eosinophilic inflammatory phenotype [49]. In phase 3 trials of benralizumab, efficacy was demonstrated in patients with baseline eosinophil counts of ≥ 300 cells/μL [80, 81].

### Add-on biologic targeting the IL-4 and IL-13 pathways

Dupilumab inhibits the IL-4 and IL-13 pathways by binding to the IL-4 receptor α-subunit and prevents the downstream activation of effectors of these cytokines. Dupilumab is approved by the FDA as add-on maintenance treatment in patients aged ≥ 6 years with moderate-to-severe, eosinophilic asthma or with oral corticosteroid-dependent asthma, regardless of blood eosinophil count [51, 82]. Dupilumab is however most efficacious in patients with blood eosinophil counts ≥ 300 cells/μL, producing a 47.7% reduction in exacerbations and a 0.32 L increase in FEV_1_ in the pivotal clinical trial [82]. In addition, post hoc analyses of the phase III trial evaluating associations between T2 biomarkers and dupilumab treatment response revealed that besides blood eosinophil counts, FeNO concentration was associated with significantly reduced exacerbations and higher FEV_1_ [83]. Given the high prevalence in patients with chronic severe asthma of airway mucus plugs showing marked increases in IL-13 gene expression [84], dupilumab might find potential success in patients with excess mucus.

### Use of biomarkers in treatment decision-making

According to the GINA strategy document, although studies are needed to identify the populations most likely to benefit from biomarker-guided treatment adjustments, such approaches may be used in patients with moderate or severe asthma managed in centers experienced in such techniques [2]. For example, a high FeNO concentration (> 50 parts per billion [ppb]) in adults is associated with ICS responsiveness [2]. FeNO is also a predictor of response to biologics; the anti-IgE biologic omalizumab yielded greater exacerbation reduction in patients with high FeNO concentration (≥ 19.5 ppb) than in patients with low FeNO (< 19.5 ppb) [66]. Further, although predictive blood eosinophil count ranges vary for different biologics, high counts are purported to predict responses to biologics targeting IL-5 and IgE [85]. Results of a meta-analysis of 16 studies of FeNO-based management and six of sputum-based management demonstrated that adjusting treatment based on FeNO levels and sputum eosinophil counts reduced the likelihood of asthma exacerbations without a significant effect on asthma control or lung function [86].

### Other options

LTRAs may have limited efficacy in broad populations but select patients might warrant a short therapeutic trial to determine whether there is substantive benefit. Use of low-dose OCSs (≤ 7.5 mg/day prednisone equivalent) should be considered as a rescue medication in adults with severe asthma despite medical therapy at GINA Step 5 [32]. With bronchial thermoplasty—a nonpharmacologic, device-based treatment—thermal energy is delivered to the airways, resulting in reduction of airway smooth muscle fibers and amelioration of asthma symptoms [87]. Bronchial thermoplasty has been evaluated in a few studies, and in a meta-analysis of three randomized controlled trials and interventional nonrandomized studies, modest improvements in asthma control and QoL measures occurred after bronchial thermoplasty [88]. In addition, evaluation of long-term outcomes of bronchial thermoplasty in patients with severe asthma indicated that 3 years after the procedure, severe exacerbations, emergency department visits, and hospitalizations significantly decreased by 45%, 55%, and 40%, respectively [89].

## Biologics and small molecules under investigation

IL-25 and IL-33 hold potential as upstream targets for the treatment of asthma. Although no biologics under investigation directly target IL-25, two anti-IL-33 antibodies, i.e., REGN3500 and ANB020 or etokimab, are under investigation [90, 91].

Various small molecules targeting specific inflammatory pathways (T2 or non-T2) are also being evaluated (Additional file 2: Table S1). The chemoattractant receptor-homologous molecule expressed on Th2 cells (CRTH2), which binds to PGD_2_ [92], is one potential key target. CRTH2, expressed on eosinophils, mast cells, and basophils, and PGD_2_ are involved in allergic inflammation [92].

Other promising targets are the STAT5/6 Src homology 2 domains. In mouse models, PM-43I—a small-molecule inhibitor of the STAT6 Src homology 2 domain that prevents recruitment to the IL-4Rα docking site and phosphorylation of Tyr641—potently inhibited STAT5- and STAT6-dependent allergic airway disease and reversed preexisting allergic airway disease [93].

Overall, these targeted therapies may play a role in individualized treatment of severe asthma; however, their application will likely be limited to patients with certain phenotypes who meet the specific criteria for use.

## Algorithm for stepwise treatment of severe asthma

The assessment and management of severe asthma is a stepwise process as outlined in the GINA strategy document, with adequate checks for adherence to treatment and correct inhaler technique at every step and with all changes in treatment. Asthma management, including medication choices, should always involve a shared decision-making process between patients and physicians. Practical considerations around dosing interval, cost, number of injections, and home versus clinic dosing, may influence choice of treatment for severe asthma.

**Fig. 3:**
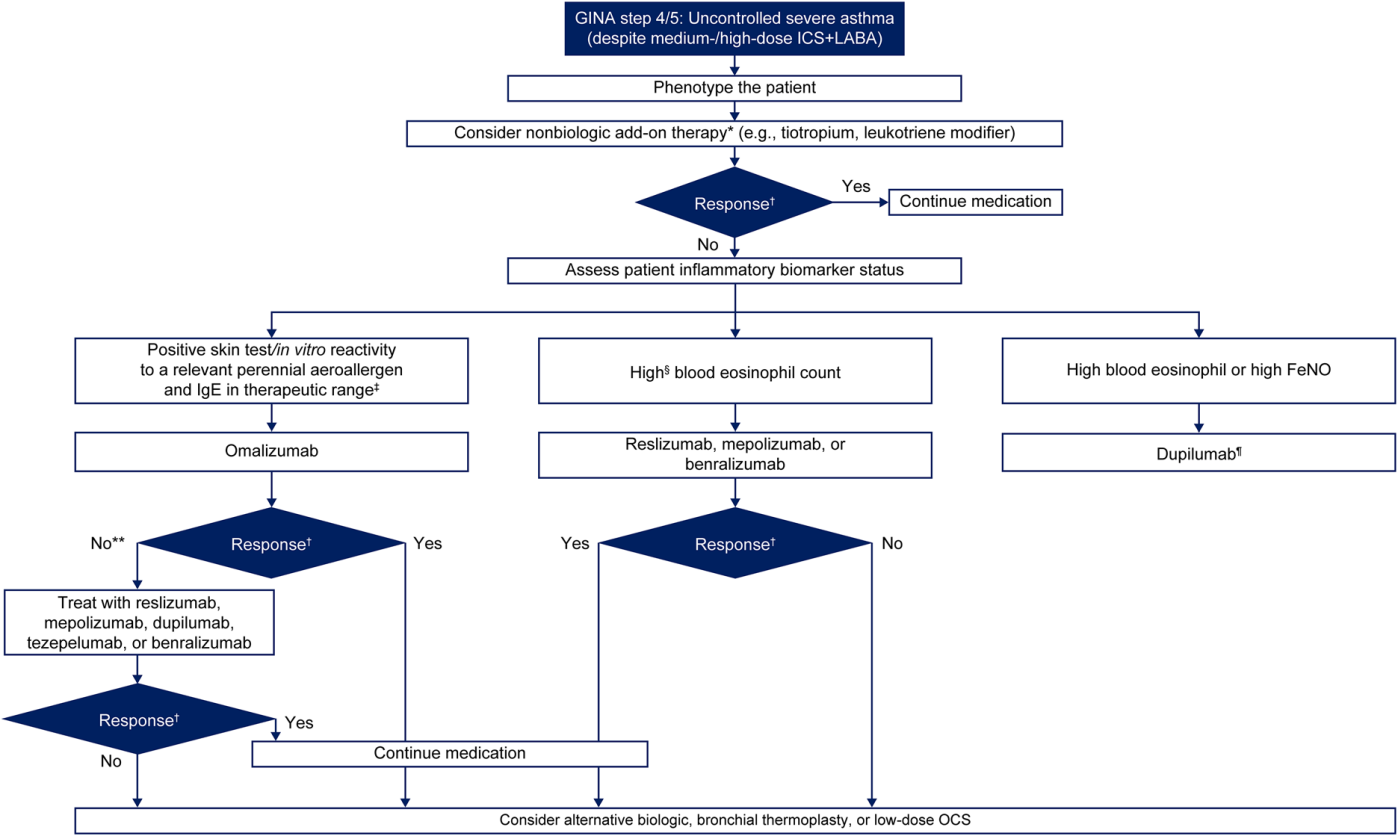
Selection of treatment options for patients with severe asthma based on clinical evaluation and biomarker levels. Biomarkers shown are not mutually exclusive. *Add-on inhaled therapy such as tiotropium may be considered before initiating biologics therapy because of the comparatively low costs associated with its use [46]. ^†^Response is defined as a reduction in exacerbations and improvement in asthma control within threshold levels. ^‡^Total IgE levels should be 30–700 IU/mL. ^§^Blood eosinophil count thresholds: reslizumab ≥ 400 µL; mepolizumab ≥ 150 cells/µL, and dupilumab and benralizumab ≥ 300 cells/µL. **Patients with high IgE levels who have blood eosinophil counts ≥ 300 cells/µL may be considered for Th2 biologic therapy. ^¶^According to GINA 2021 recommendations [2], potential predictors of good asthma response include increasing baseline levels of blood eosinophils and FeNO [82]. *FeNO* fractional exhaled nitric oxide, *FDA* Food and Drug Administration, *GINA* Global Initiative for Asthma, *ICS* inhaled corticosteroids, *Ig* immunoglobulin, *LABA* long-acting β_2_-agonist, *LTRA* leukotriene receptor antagonist, *OCS* oral corticosteroid, *Th* T helper

Although biologics are typically recommended for patients with severe, uncontrolled disease, high costs, parenteral administration at regular intervals, and regular monitoring may prove to be barriers to their use. In the current treatment landscape, a common dilemma that physicians face is whether to proceed to treatment with add-on biologics directly or to add a LAMA. We propose a provisional algorithm, based on available data, for the treatment of severe asthma, that builds upon the recommendations of GINA [2, 32] (Fig. 3; see Additional file 1: Video S1).

The complete benefits of non-biologic inhaled therapies are perhaps not being fully considered before the switch to biologics. We recommend that inhaled therapies such as tiotropium should be considered before moving to a biologic therapy as they may prove efficacious and achieve control in a range of patients (i.e., of various age groups, across asthma severities, and independent of their eosinophil counts) [45]. Notably, tiotropium was approved in 2014 (2017 for ages 6–11) when most pivotal trials on biologics were conducted [71, 73–76] or a minority of patients were on LAMAs at the time of entry into these trials [70, 80, 81]. Furthermore, treatment recommendations do not strongly advocate the use of LAMAs as add-on therapy [2, 29].

If all recommended inhaled and oral therapies are ineffective, phenotyping is recommended, and biomarker screening tests should be considered to determine the most appropriate step-up therapy using a shared decision-making approach with the patient. In the absence of direct clinical comparisons of biologics, the choice of biologic may be determined by the physician based on specific biomarkers underlying patient asthma phenotypes. Bronchial thermoplasty should be considered in appropriate patients as well [32].

## Conclusions

Asthma is a chronic disease comprising multiple clinical and inflammatory phenotypes. Besides medium- or high-dose ICS + LABA, tiotropium and several biologics, tailored toward specific inflammatory phenotypes, are approved as add-on therapies for treatment of severe asthma. Before considering the use of biologics, add-on inhaled therapies, such as LAMAs, may provide scope for improvement in asthma control owing to their comparatively low cost. Notably, the on-going development of new biologics and small molecules may pave the way for targeted treatments of patients with severe asthma with an appropriate phenotype.

## Supplementary Information

**Additional file 1: Video S1.** Overview of the pathophysiology of asthma, mechanism of action of available inhaled therapies and biologics, and the proposed treatment algorithm for severe asthma.

**Additional file 2: Table S1.** Small molecules in asthma under investigation.

## Data Availability

Not applicable.

## Abbreviations

CRTH2: Chemoattractant receptor-homologous molecule expressed on Th2 cells
FDA: Food and Drug Administration
FeNO: Fractional exhaled nitric oxide
FEV_1_: Forced expiratory volume in 1 s
GINA: Global Initiative for Asthma
Ig: Immunoglobulin
ILC2: Innate lymphoid cell type 2
ICS: Inhaled corticosteroids
IL: Interleukin
LABA: Long-acting β_2_-agonist
LAMA: Long-acting muscarinic antagonist
OCS: Oral corticosteroids
PGD_2_: Prostaglandin D_2_
ppb: Parts per billion
QoL: Quality of life
STAT: Signal transducer and activator of transcription
Th2: T-helper type 2
TSLP: Thymic stromal lymphopoietin
T2: Type 2
