# Supplementary material for: The new era of add-on asthma treatments: where do we stand?
Source: Allergy Asthma Clin Immunol. 2022 May 21;18:42. doi: 10.1186/s13223-022-00676-0 (PMC9124422; doi:10.1186/s13223-022-00676-0)
Supplement: Supplementary file 2 — Additional file 2: Table S1. Small molecules in asthma under investigation. [file 13223_2022_676_MOESM2_ESM.docx]

**Additional file 2**

Table S1: Small molecules in asthma under investigation

| **Category** | **Drug** | **Route of administ-ration** | **Clinical stage** | **Source** |
| --- | --- | --- | --- | --- |
| **LAMA** | Umeclidinium bromide (+ fluticasone furoate) | Inhalation | Phase 2b | https://clinicaltrials.gov/ct2/show/NCT03012061?term=NCT03012061&draw=2&rank=1 |
|  | Umeclidinium bromide (+ fluticasone furoate + vilanterol) |  | Phase 3 | <https://clinicaltrials.gov/ct2/show/NCT02924688?term=GSK573719+in+Combination+With+Fluticasone+Furoate&draw=3&rank=7> |
|  | Glycopyrronium (glycopyrrolate) |  | Phase 2/3 | https://clinicaltrials.gov/ct2/show/NCT03358147?term=PT001&draw=2&rank=9 |
| **Leukotriene receptor antagonist/leukotriene inhibitor** | Gemilukast | Oral | Phase 2 | <https://clinicaltrials.gov/ct2/show/NCT01536041?term=Gemilukast&cond=asthma&rank=2> |
|  | GSK2190915 |  |  | <https://clinicaltrials.gov/ct2/show/NCT01156792?term=GSK2190915&cond=asthma&rank=2> |
| **CRTH2 antagonist** | GB001 | Oral | Phase 2 | <https://clinicaltrials.gov/ct2/show/NCT03683576> |
| **PDE4 inhibitor** | Roflumilast | Oral | Phase 2 | <https://clinicaltrials.gov/ct2/show/NCT01765192?term=Roflumilast&cond=ASTHMA&rank=1> |
|  | CHF6001 | Inhalation |  | <https://clinicaltrials.gov/ct2/show/NCT01689571?term=CHF6001&cond=Asthma&rank=1> |
| **Dual PDE3 and PDE4 inhibitor** | RPL554 | Inhalation | Phase 2 | <https://clinicaltrials.gov/ct2/show/NCT02427165?term=RPL554&cond=ASTHMA&rank=1> |
| **Protein kinase inhibitor** | Imatinib | Oral | Phase 2 | <https://clinicaltrials.gov/ct2/show/NCT01097694?term=Imatinib&cond=Asthma&rank=1> |
|  | Masitinib |  | Phase 3 | <https://clinicaltrials.gov/ct2/show/NCT03771040?term=Masitinib&cond=Asthma&rank=1> |
| **Selective glucocorticoid receptor modulator** | AZD7594 |  | Phase 2 | <https://clinicaltrials.gov/ct2/show/NCT03622112?term=AZD7594&cond=Asthma&rank=2> |
| **Macrolide** |  |  |  |  |
|  | Roxithromycin | - | Pre-clinical | [1] |
|  | CSY0073 |  |  | [2] |
|  | MAC5 |  |  | [3] |
| **Statin** | Atorvastatin | Oral | Phase 2 | <https://clinicaltrials.gov/ct2/show/NCT00126048?term=Atorvastatin&cond=Asthma&rank=2> |
| **Novel β_2_-adrenoceptor agonist** | MN-221 | Intravenous | Phase 2 | https://clinicaltrials.gov/ct2/show/NCT00838591?term=MN-221&cond=ASTHMA&draw=2&rank=1 |

*CRTH2* chemoattractant receptor-homologous molecule expressed on Th2 cells, *LAMA* long‑acting muscarinic receptor antagonist, *PDE* phosphodiesterase, *Th* T helper.

References

1. Pei QM, Jiang P, Yang M*,* Qian XJ, Liu JB, Kim SH*.* Roxithromycin inhibits VEGF-induced human airway smooth muscle cell proliferation: Opportunities for the treatment of asthma. Exp Cell Res. 2016;347:378–384
2. Balloy V, Deveaux A, Lebeaux D*,* Tabary O, Le Rouzic P, Ghigo JM, et al*.* Azithromycin analogue CSY0073 attenuates lung inflammation induced by LPS challenge. Br J Pharmacol. 2014;171:1783–1794
3. Porter JD, Watson J, Roberts LR*,* Gill SK., Groves H, Dhariwal J, et al*.* Identification of novel macrolides with antibacterial, anti-inflammatory and type I and III IFN-augmenting activity in airway epithelium. J Antimicrob Chemother. 2016;71:2767–2781
